# Childhood maltreatment’s influence on the dynamic course of depression: symptom trajectories during inpatient treatment and after discharge

**DOI:** 10.1017/S0033291725000984

**Published:** 2025-05-02

**Authors:** Janette Ratzsch, Maike Richter, Rogério Blitz, Lejla Colic, Lara Gutfleisch, Janik Goltermann, Marius Gruber, Benjamin Straube, Nina Alexander, Hamidreza Jamalabadi, Frederike Stein, Katharina Brosch, Florian Thomas-Odenthal, Paula Usemann, Lea Teutenberg, Jonathan Repple, Bernhard T. Baune, Martin Walter, Igor Nenadić, Tilo Kircher, Udo Dannlowski, Nils Opel

**Affiliations:** 1 Department of Psychiatry and Psychotherapy, Jena University Hospital, Jena, Germany; 2 Center for Intervention and Research on Adaptive and Maladaptive Brain Circuits Underlying Mental Health (C-I-R-C), Jena-Magdeburg-Halle, Germany; 3 German Center for Mental Health (DZPG), Site Jena-Magdeburg-Halle, Germany; 4 Institute for Translational Psychiatry, University of Münster, Münster, Germany; 5 Department for Psychiatry, Psychosomatic Medicine and Psychotherapy, University Hospital Frankfurt, Goethe University, Frankfurt am Main, Germany; 6 Department of Psychiatry and Psychotherapy, Philipps-Universität Marburg and University Hospital Marburg, UKGM, Marburg, Germany; 7 Center for Mind, Brain and Behavior (CMBB), Marburg, Germany; 8 Institute of Behavioral Science, Feinstein Institutes for Medical Research, Manhasset, NY, USA; 9 Department of Psychiatry, Melbourne Medical School, The University of Melbourne, Melbourne, VIC, Australia; 10 The Florey Institute of Neuroscience and Mental Health, The University of Melbourne, Parkville, VIC, Australia; 11 Department of Psychiatry, University of Münster, Münster, Germany; 12 Goethe University Frankfurt, Cooperative Brain Imaging Center – CoBIC, Frankfurt, Germany

**Keywords:** childhood maltreatment, clinical routine samplelong-term course, major depressive disorder, relapse, symptom trajectories

## Abstract

**Background:**

Many studies have highlighted the detrimental effect of childhood maltreatment (CM) on depression severity and the course of illness in major depressive disorder (MDD). Yet our understanding of how CM influences the dynamic symptom change throughout a patient’s trajectory remains limited. Hence, we investigated the impact of CM on depression severity in MDD with a focus on various treatment phases during inpatient treatment and after discharge (1 or 2 years later) and validated findings in a real-world setting.

**Methods:**

We used longitudinal data from a cohort study sample (*n* = 567) and a clinical routine sample (*n* = 438). CM was measured with the Childhood Trauma Questionnaire (CTQ), and depression severity was assessed using Beck’s Depression Inventory (BDI). The long-term clinical trajectory was assessed using the Life Chart Interview.

**Results:**

Our analyses revealed that CM significantly increased depression severity before, during, and after inpatient therapy in both samples. Although CM was associated with higher depression severity at the beginning of inpatient treatment and lower remission rates upon discharge, no discernible impact of CM was evident on the relative change in symptoms over time during inpatient treatment. CM consistently predicted higher relapse rates and lower rates of full remission after discharge during long-term follow-up in both samples.

**Conclusions:**

Our findings affirm the link between CM and the development of more severe and persistent clinical trajectories within real-world clinical settings. Furthermore, conventional psychiatric treatments may not lead to comparable outcomes for individuals with a history of CM, underscoring the necessity for tailored therapeutic interventions.

## Implications

Childhood maltreatment experiences should be assessed and considered during treatment for depressive episodes as treatment as usual may not effectively alleviate depressive symptoms in maltreated individuals. Thus, there is a need to explore effective treatments for individuals with CM.

## Public significance statement

The Childhood Trauma Questionnaire holds vital potential for clinicians to identify patients at high risk of enduring persistent depression due to childhood maltreatment. Future research endeavors should focus on developing and investigating tailored treatment programs for this vulnerable subgroup, offering the promise of enhanced mental health care and improved outcomes.

## Introduction

Major depression is a common and debilitating illness that often has a recurrent and progressive course (Richards, 2011). Its heterogeneous nature, characterized by variations in age of onset and symptom severity, has significant implications for disease trajectory and treatment response (Carter et al., 2012; Heun, Kockler, & Papassotiropoulos, 2000). Identifying factors that predict the risk for recurrent and persistent depressive episodes, as well as for treatment resistance is essential due to the substantial health impact and economic burden associated with poor longitudinal outcomes (Nanni, Uher, & Danese, 2012).

Childhood maltreatment (CM) is recognized as a prominent risk factor for psychiatric disorders, which even led to suggestions that individuals with CM should be regarded as a distinct group (Teicher & Samson, 2013). CM has been associated with increased vulnerability to depression (Lippard & Nemeroff, 2020). Its prevalence in patients with severe mental disorders emphasizes its importance in clinical care (Teicher, Gordon, & Nemeroff, 2022). Individuals with persistent depressive disorder often report higher CM experiences than non-chronic patients with depressive disorders, and depression severity has been correlated with experiences of emotional abuse and neglect (Struck et al., 2020). Moreover, maltreated individuals have a higher risk for suicide ideation and behaviors and poorer treatment responses, with higher rates of chronic or treatment-resistant depression (Nelson, Klumparendt, Doebler, & Ehring, 2017; Teicher & Samson, 2013).

Previous studies have highlighted the significance of CM in predicting unfavorable disease courses and treatment outcomes in depression (Nanni, Uher, & Danese, 2012). However, there are still significant gaps in existing research as there is a lack of comprehensive data that examines the course or dynamics of depressive symptoms, both in the short and long term. Observation of periods rather than distinct time points is on demand to get a whole perspective and to identify different pathways of depressive course after treatment for a better understanding and prediction of treatment response. For instance, emotional abuse has been found to affect the risk of depression onset with an effect size that is twice as high as compared to physical abuse (Norman et al., 2012). Moreover, the significant influence of CM is evident in its capacity to moderate treatment responses negatively across various mental health domains (Bruce, Heimberg, Goldin, & Gross, 2013; Euler et al., 2019; Scott, McLaughlin, Smith, & Ellis, 2012; Thomas, Höfler, Schäfer, & Trautmann, 2019). Additionally, recent research indicates that altering the subjective perception of CM may enhance the long-term trajectory of emotional disorders (Danese & Widom, 2023).

In the realm of therapy for depressive disorders, where inpatient treatment is prevalent, the impact of CM on treatment response deserves heightened scrutiny. Existing research indicates that individuals with depression and CM history often exhibit insufficient response to treatment, subsequently facing elevated risks of recurring and persistent depressive episodes (Lippard & Nemeroff, 2020). However, these findings await validation within real-world settings due to several crucial limitations. One primary concern lies in the characteristics of previous research samples, which often suffered from selection bias, reliance on artificial or controlled conditions, and, hence, questionable generalizability. A meta-analysis on treatment efficacy in persons with major depressive disorder and CM history showed, most studies had a moderate to high risk of bias (Kuzminskaite et al., 2022). For instance, limitations were a high rate of non-participation among women randomly assigned to interpersonal psychotherapy (Toth et al., 2013) or a sample of patients from academic centers (Schramm et al., 2017).

These shortcomings underline the need for samples derived from real-world settings, characterized by their representativeness and heterogeneity within the diverse spectrum of patients undergoing inpatient treatment. Such samples offer the advantage of reflecting the complexities of everyday life and enhancing the external validity of research findings. Moreover, details regarding depression course and recovery – such as recurrence frequency, remission outcomes, and chronicity – can potentially inform inpatient therapeutic strategies (Greer, Kurian, & Trivedi, 2010).

This study aims to investigate the influence of CM on symptom trajectories during inpatient treatment and after discharge of individuals with major depressive disorder (MDD) and to validate previous findings (Lippard & Nemeroff, 2020) in a real-world setting. Hence, we extensively investigated both a clinical routine sample and a cohort study sample. Based on prior findings, the following hypotheses are formulated:

1. We expect patients with CM to exhibit a prolonged illness history alongside more pronounced and severe depressive symptoms at the start of inpatient treatment.
2. Further, we predict that patients with CM history exhibit smaller improvement during inpatient treatment when compared with patients without CM.
3. Last, we assume that patients with CM show higher rates of relapse and lower rates of remission during 1- and/or 2-year follow-up periods after discharge from inpatient treatment.

## Methods

### Participants and design

This investigation utilized data sets from two distinct samples: a clinical routine sample (CRS, *n* = 438) and a cohort study sample (CoSS, *n* = 567). In total, we included *n* = 1005, with 55.02% women (age mean 38.35 ± 14.73). All patients meeting the criteria for inpatient treatment of MDD at baseline, including MDD with psychotic features, were included, while those with comorbid psychotic and substance-related disorders were excluded to ensure consistency.

The CRS data were sourced from the IZKF SEED 11/18 study, a longitudinal naturalistic within the scope of inpatient psychiatric treatment (Richter et al., 2020).

The data utilized in the CoSS were derived from two comparable neuroimaging studies – the ongoing Marburg-Münster Affective Disorders Cohort Study (MACS consortium) (Kircher et al., 2019) and the Münster Neuroimaging Cohort Study (Opel et al., 2019; Redlich et al., 2016; Zaremba et al., 2018).

Patients in both samples received standard inpatient treatment with a focus on depression treatment and not specifically focusing on CM. The treatment consisted of pharmacotherapy and psychotherapy with CBT (cognitive behavioral therapy) with one 50-min one-on-one therapy session per week with a clinical psychologist or psychiatrist. Depending on individual treatment plans, inpatients also participated in group therapy and complementary treatments (e.g., social therapy, art therapy) led by psychologists, nurses, physiotherapists, or social workers.

Across both sample groups, we carried out a series of assessments, including retrospective evaluations, baseline appraisals during their inpatient therapy initiation, and follow-up evaluations. Specifically, the CRS received bi-weekly assessments throughout their therapy. Furthermore, follow-up assessments were conducted 1 year after treatment completion for the CRS and 2 years for the CoSS. For more details on the samples, please refer to supplementary methods.

### Measures

The German version of the Childhood Trauma Questionnaire (CTQ) (Wingenfeld et al., 2010) was used to evaluate CM, including a sum score and subscales for emotional neglect, physical neglect, emotional abuse, physical abuse, and sexual abuse (Bernstein et al., 1994).

In retrospective clinical analyses, we utilized the number of hospitalizations and age at the initial psychiatric or psychotherapeutic treatment. Depression severity progression was measured using the cumulative score from the Beck Depression Inventory (BDI, Beck et al., 1961), the Hamilton Depression Scale (HAMD-17) (Hamilton, 1967), and the Global Assessment of Functioning Scale (GAF) (Hall, 1995) for an overall assessment of functioning along a continuum from psychological distress to well-being.

Furthermore, exclusively within the CRS, we incorporated three additional BDI measurements at approximately 14-day intervals. These scores were utilized to investigate response rates, defined as a ≥50% reduction in symptoms from baseline (Keller, 2003), and remission rates as a BDI score of ≤12 (Riedel et al., 2010).

The Life Chart Interview, a rater-based measure was employed to identify depressive episodes and to evaluate relapse, establish full remission and distinct symptom trajectories within the follow-up period.

For more details on measures, please refer to supplementary methods.

### Statistical analyses

Analyses were conducted in R version 4.3.0 and IBM SPSS Version 29.

Due to differences in the recruitment strategy for both samples, it was hypothesized that systematic disparities may exist. Consequently, comparative analyses were conducted between the two sample groups. These individual analyses encompassed various factors, including age, CM levels, depression severity assessed through BDI and HAMD-17, general functionality measured via GAF, age at the initiation of psychiatric or psychotherapeutic treatment, and the number of hospitalizations prior to the commencement of inpatient therapy. Two-sample t-tests were employed for continuous variables, while for dichotomous variables such as gender, comorbidities, and relapse (yes/no), χ^2^-tests were performed.

#### Childhood maltreatment’s Influence on illness history and depression severity prior to inpatient treatment

First, linear regression models were used to test whether the sum score of the CTQ was an overall predictor for illness history and the severity of depressive symptoms at the start of inpatient treatment. In each regression model, age and gender were included as covariates and within further analysis site in the CoSS. Number of hospitalizations, age at first psychiatric or psychotherapeutic treatment, and BDI were entered as the outcome variables. Analyses were performed in both the CRS and the CoSS.

#### Childhood maltreatment’s influence on symptom trajectories during inpatient treatment

To address our second hypothesis, we employed a linear mixed model (LMM) with repeated measurements exclusively in the CRS, where data were available during inpatient treatment. Analyses were carried out in R with the package “lmer” for linear mixed models (Kuznetsova, Brockhoff, & Christensen, 2017). The outcome variable of interest was the BDI score measured at baseline and three additional time points (2, 4, and 6 weeks into therapy, time intervals measured in days). These specific time intervals were selected to provide a sequence of four continuous observations and to ensure an adequately sized patient subgroup (*n* = 98) in the CRS with complete data across these time points.

In the first model, the predictors included the CTQ and time to analyze the main effects of CTQ and time. A significant effect of CTQ would indicate an overall impact on BDI scores over the symptom trajectories during inpatient treatment. Age and gender were added as covariates. The LMM included both random intercept and random slope components.

In the second model, the predictors included the CTQ, time, and additionally the interaction of CTQ × time. A significant interaction of CTQ x time would suggest differential patterns of symptom trajectories during inpatient treatment based on CM levels.

To further validate our findings, we employed logistic regression models to assess response and remission rates within the CRS.

In each logistic regression model for response and remission rates, we included CTQ as a fixed factor and controlled for age, gender, and, in a secondary step, baseline BDI scores.

#### Childhood maltreatment’s influence on symptom trajectories after discharge

Third, we used logistic regression models to examine the impact of CM on relapse and remission rates in the follow-up period as important indicators of symptom trajectories after discharge. Each regression model included age and gender as covariates and within further analysis site in the CoSS. Univariate variance analysis was employed to identify variations in CM values across specific symptom trajectories during the 1–2 years follow-up period after discharge. These analyses were conducted for a 1-year follow-up period in the CRS and a 2-year follow-up in the CoSS.

All models were considered significant at the *p* < 0.05 and corrected for multiple comparisons.

## Results

### Sample

Our study involved 438 participants in the clinical CRS and 567 participants in the CoSS. Follow-up assessments were conducted with 246 participants (56% female, age 40.5 ± 16.94) in the CRS after 1 year and 441 participants (56% female, age 38.72 ± 13.13) in the CoSS after 2 years. We observed substantial differences in several sociodemographic and clinical features between the samples (Table 1).

**Table 1.** Overview of descriptive sociodemographic and clinical data

	CRS (*n* = 438)	CoSS (*n* = 567)	*t*/*X*^2^	*p*
Age (mean ± S.D.)	39.34 ± 16.84 (18–81a)	37.59 ± 12.83 (18–65a)	1.87	<.001
Gender (M/F)	199/239	253/314	0.07	.797
Age at first psychiatric or psychotherapeutic treatment	30.03 ± 16.03	31.12 ± 12.44	−2.64	<.001
Number of hospitalizations (mean ± S.D.)	3.12 ± 4.35	2.38 ± 2.46	1.81	<.001
Length of current psychiatric treatment, mean ± S.D. (range: min–max, in days)	46.243 ± 24.003 (4–127)			
Childhood trauma severity (min 5 to max 25 for subtypes of trauma)				
All trauma, mean ± S.D. (range: min–max)	47.46 ± 16.80 (25–111)	46.88 ± 16.88 (25–119)	0.52	.789
Emotional neglect, mean ± S.D. (range: min–max)	13.84 ± 5.78 (5–25)	13.76 ± 5.61 (5–25)	0.21	.341
Emotional abuse, mean ± S.D. (range: min–max)	11.67 ± 5.75 (5–25)	11.28 ± 5.39 (5–25)	1.11	.055
Physical neglect, mean ± S.D. (range: min–max)	11.67 ± 5.75 (5–25)	8.23 ± 3.14 (5–22)	11.92	<.001
Physical abuse, mean ± S.D. (range: min–max)	7.18 ± 3.59 (5–25)	7.24 ± 3.75 (5–24)	−0.33	.284
Sexual abuse, mean ± S.D. (range: min–max)	6.68 ± 3.68 (5–25)	6.45 ± 3.67 (5–25)	0.95	.348
Beck’s depression Inventory-II (min 0 to max63 for sum score)				
Baseline (mean ± S.D.)	25.23 ± 11.27	25.16 ± 10.17	0.15	.001
After 2 weeks (mean ± S.D.)	21.17 ± 11.57			
After 4 weeks (mean ± S.D.)	20.55 ± 12.56			
After 6 weeks (mean ± S.D.)	19.45 ± 12.03			
Discharge (mean ± S.D.)	13.57 ± 11.35			
Follow Up after 1 (CRS)/2 (CoSS) years (mean ± S.D.)	18.80 ± 13.03	15.53 ± 12.18	2.65	.290
HAMD–17 score				
Baseline (mean ± S.D.)	17.55 ± 6.91	16.93 ± 7.79	1.25	<.001
Follow Up (mean ± S.D.)	11.34 ± 7.99	8.01 ± 6.98	5.10	.004
GAF score				
Baseline (mean ± S.D.)	53.67 ± 10.15	53.86 ± 9.28	−0.29	.040
Follow up (mean ± S.D.)	57.42 ± 11.28	68.68 ± 14.96	1.41	<.001
Comorbidities (%)	42.92%	49.91%	6.64	.036
Relapse (%)	74.17%	58.33%	15.59	<.001

Abbreviations: CRS, Clinical Routine Sample; CoSS, Cohort Study Sample; HAM-D, Hamilton Rating Scale for Depression; GAF, General Assessment of Functioning Scale.

### Childhood maltreatment’s influence on illness history and depression severity prior to inpatient treatment

Linear regression models revealed a significant influence of CM on key indicators of prolonged illness history within both groups. Specifically, CM was associated with a higher number of hospitalizations (CRS: Beta coefficient (*B*) = 0.04, Standard error (*SE*) = 0.01, *p* < 0.0001, CoSS: *B* = 0.036, *SE* = 0.006, *p* < 0.0001) and a younger age at first psychiatric or psychotherapeutic treatment (CRS: *B* = −0.091, *SE* = 0.029, *p* = 0.002, CoSS: *B* = −0.139, *SE* = 0.019, *p* < 0.0001) (see Table S1 in the supplementary material).

Furthermore, the CTQ score was positively associated with the BDI at the start of inpatient treatment (CRS: *B* = 0.237, *SE* = 0.031, *p* < 0.0001, CoSS: *B* = 0.183, *SE* = 0.025, *p* < 0.0001) (Supplementary Table S1). Findings were confirmed by HAMD-17 and GAF results (Supplementary Table S2), with consistent outcomes in CoSS, controlling for the site (Supplementary Table S3).

These associations were found consistently with all CTQ subscales (see Supplementary Table S4). Controlling for the site in CoSS, the outcomes remained consistent (see Supplementary Table S5).

### Childhood maltreatment’s influence on symptom trajectories during inpatient treatment

Results of linear mixed models within the CRS in Model A indicated a significant decline in BDI values over time (*B* = −0.159, *SE* = 0.022, *p* = <0.001) (see Supplementary Table S6). Additionally, the CTQ score predicted higher overall BDI values (*B* = 0.177, *SE* = 0.065, *p* = 0.008).

Notably, the random intercept demonstrated substantial variance (σ^2^ = 86.316, *SD* = 9.291), indicating considerable differences between individuals. Conversely, random effects within time intervals were relatively small (*σ*^2^ = 0.027, *SD* = 0.165), suggesting consistency in observations across time intervals.

Results in Model B showed that the interaction between CTQ score and time did not yield a significant impact on BDI values (*B* = −0.002, *SE* = 0.001, *p* = 0.152) (see model B Supplementary Table S6). Notably, only in the CTQ subscale of physical abuse, the interaction effect was significant (see Supplementary Table S7).

**Figure 1.**
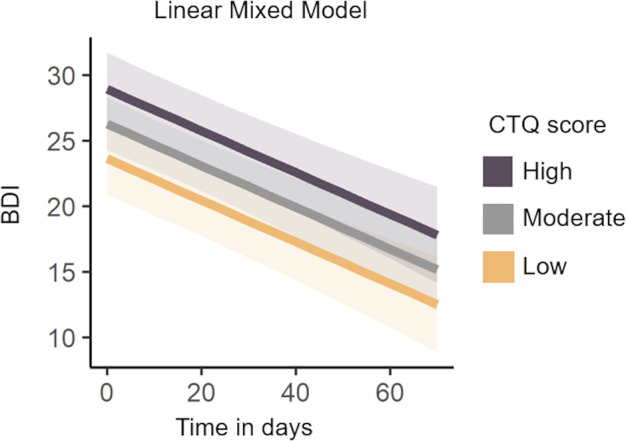
Change in BDI values over time during inpatient treatment depending on the severity of CM. *Note:* CTQ = Childhood Maltreatment Questionnaire, BDI = Beck’s Depression Inventory. CTQ values are divided into three distinct groups to illustrate the absence of an interaction effect.

In summary, while individuals with higher levels of CM initiated therapy with elevated BDI values, symptom reduction over time was not moderated by CM (see Figure 1). Regarding the results of symptom trajectories during inpatient treatment, it seems that significant differences in BDI scores related to CM persist at discharge of inpatient treatment.

The findings from the LMM were supported by the results for response and remission rates. After the initial 2-week period, the CTQ score exhibited a significant negative impact on response rates (*B* = −0.004, *SE* = 0.001, *p* < 0.001), even when accounting for baseline depression severity (BDI Baseline) (see Supplementary Table S8).

However, consistent with the abovementioned results, by the end of 4 weeks, CTQ score no longer exerted a significant influence on response rates (*B* = −0.001, *SE* = 0.001, *p* = 0.659). When adjusting for BDI at baseline, the previously observed significant influence of CM on response rates is removed, while the BDI baseline retains its significant negative impact on response rates (CTQ: *B* = 0.001, *SE* = 0.002, *p* = 0.491; BDI baseline: *B* = −0.010, *SE* = 0.003, *p* = 0.002).

At the conclusion of inpatient treatment, remission was achieved by 141 out of 244 patients (57.8%). CTQ demonstrated a significant negative impact on remission rates (*B* = −0.006, *SE* = 0.002, *p* = 0.004). In contrast, when accounting for BDI at baseline, CM no longer exhibited a significant influence on remission rates (CM: *B* = −0.0004, *SE* = 0.002, *p* = 0.825; BDI Baseline: *B* = −0.020, *SE* = 0.003, *p* < 0.0001) (Supplementary Table S8). Findings were consistent on remission rates concerning HAMD-17 (Supplementary Table S9). Additionally, CM exhibited no significant impact on the length of inpatient treatment (*B* = −0.014, *SE* = 0.096, *p* = 0.881) (see Supplementary Table S1). The length of inpatient treatment ranged from 4 to 127 days, with a mean value of 46.243 days (*SD* = 24.003).

In summary, the outcomes derived from the linear mixed model analysis align with the findings from logistic regression analyses on response and remission rates during inpatient treatment.

### Childhood maltreatment’s influence on symptom trajectories after discharge

**Figure 2.**
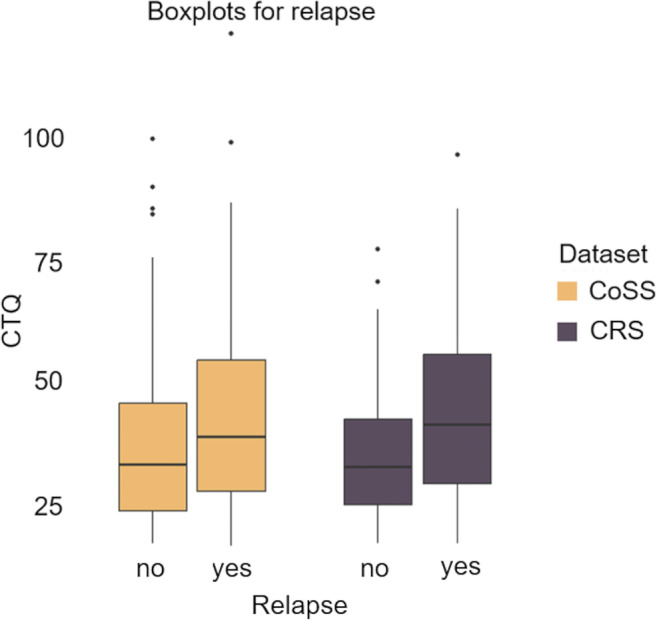
CTQ score by relapse during one-year follow-up interval in the CRS and two-year follow-up interval in the CoSS. *Note:* CTQ = Childhood Maltreatment Questionnaire, CRS = Clinical Routine Sample, CoSS = Cohort Study Sample.

Individuals with higher CTQ scores were significantly more likely to *relapse* during the follow-up period across both samples (CRS: *B* = 0.005, *SE* = 0.002, *p* = 0.002; CoSS: *B* = 0.007, *SE* = 0.002, *p* = 0.003) (see Figure 2 and Supplementary Table S10). Notably, the effect sizes associated with all CTQ subscales exhibited a consistent pattern (see Supplementary Table S11).

**Figure 3.**
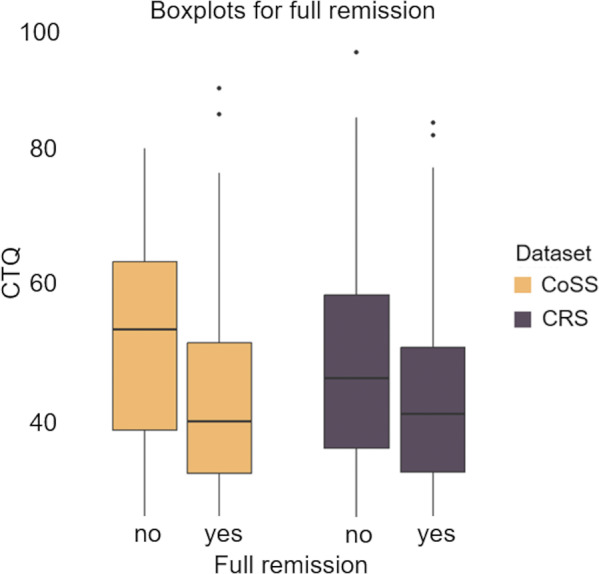
CTQ score by achieving full remission during one-year follow-up interval in the CRS and two-year follow-up interval in the CoSS. *Note:* CTQ = Childhood Maltreatment Questionnaire, CRS = Clinical Routine Sample, CoSS = Cohort Study Sample.

Subsequent analyses pertain to distinct symptom trajectories. The CTQ score was a predictive factor for determining whether patients achieved *full remission* during the follow-up period in both samples (CRS: *B* = −0.005, *SE* = 0.002, *p* = 0.017; CoSS: *B* = −0.008, *SE* = 0.002, *p* < 0.001) (see Figure 3 and Supplementary Table S10). Controlling for the site in CoSS, the outcomes remained consistent (Supplementary Table S12).

Regarding all symptom trajectories, we observed a consistent pattern of higher CTQ values in patients with more severe, more frequent, or prolonged periods of depressive symptoms (Supplementary Table S13). Moreover, CTQ score emerged as a significant predictor for BDI during the follow-up intervals and the duration of depressive episodes during the follow-up period (Supplementary Table S14). Additionally, the robustness of these outcomes is underscored by consistent findings in the CoSS, even after controlling for site variability (Supplementary Table S3).

More details on these results can be found in supplementary results.

## Discussion

Our investigation focused on the influence of CM on individuals diagnosed with MDD across various phases of their treatment journey and study settings, encompassing a representative real-world setting.

The results in support of the first hypothesis demonstrated consistent associations with well-established findings regarding CM and psychiatric history (Behr Gomes Jardim et al., 2018; Benjet, Borges, & Medina-Mora, 2010; Green et al., 2010; Lippard & Nemeroff, 2020; Volgenau, Hokes, Hacker, & Adams, 2022). Our findings implicate that individuals diagnosed with MDD and a history of CM tend to exhibit an extended history of illness alongside more severe depressive symptoms. These associations extend to the GAF, which not only reflects depression but also the severity of comorbidities. Our results further indicate that CM affects the overall level of function across multiple time points throughout the course of illness. Consequently, CM should be recognized as a critical factor that affects mental health beyond the depressive symptoms.

We did not observe a significant influence of CM in relative symptom change during overall symptom trajectories of inpatient treatment. This is surprising because we hypothesized a smaller improvement during inpatient treatment in patients with CM history. Additionally, CM did not moderate the length of inpatient treatment in the CRS sample. Nonetheless, at the conclusion of inpatient treatment, those with experiences of CM exhibited lower rates of remission. However, the positive effect of treatment is not sufficient to alleviate the increased levels of depressive symptoms at the initiation of inpatient for MDD patients with CM history. We therefore conclude that novel and more effective treatment approaches, as previously recommended by Teicher and Samson (2013), are needed to address the higher rates of depressive symptoms observed at the initiation and completion of treatment in CM patients.

However, upon close examination of CM subscales, only the impact of physical abuse on depression severity significantly diminished during inpatient treatment. This may be attributed to factors such as intensive care, heightened safety, therapeutic surroundings, and reduced external stressors. The resulting sense of safety can lead to a reduction in anxiety and hypervigilance, prevalent in abuse survivors (Malinosky-Rummell & Hansen, 1993), consequently influencing depression severity (Fava et al., 2004). Patients with depression and a history of physical abuse might find greater benefit in an inpatient setting.

In contrast to physical abuse, sexual abuse did not appear to have any overall impact on symptom trajectories during inpatient treatment. This observation could potentially be attributed to limitations in statistical power due to a relatively small number of patients reporting sexual abuse and to a wide range of responses to therapy in this subgroup (Saywitz, Mannarino, Berliner, & Cohen, 2000).

Various studies have reported varied findings regarding the impact of CM on improvements during therapy. Shirk et al. reported the negative association of trauma history with symptom change and treatment response (Shirk, Kaplinski, & Gudmundsen, 2009). Another study found that trauma history moderated treatment response (Lewis et al., 2010). Focusing on different treatment phases, childhood trauma predicted the antidepressant treatment outcome only during the early treatment phase (Ju et al., 2020), which mirrors our findings. Moreover, our results align with recent meta-analysis results, indicating similar trajectories of improvement in MDD patients with and without CM following pharmacological and psychotherapeutic treatment (Kuzminskaite et al., 2022).

In our investigations on symptom trajectories after discharge, we found that the lasting impact of CM on depression severity persisted in both sample groups even 1 and 2 years after inpatient treatment. CM emerged as a predictor for relapse rates, the duration of depressive episodes, and full remission attainment during follow-up, albeit with modest effect sizes. Moreover, the CTQ subscales exhibited effect sizes and directional patterns that were uniform in both samples.

Based on these findings, it is crucial to enhance post-inpatient therapy for CM patients not achieving full remission during inpatient treatment. A comprehensive approach to post-inpatient maintenance therapy and relapse prevention is essential, prioritizing ongoing, patient-specific care to improve long-term mental health and recognize individual symptom trajectories.

It should furthermore be noted that patients in the present study exclusively received standard psychotherapy for depression. Improvement in depressive symptoms in the initial treatment phase of standard psychotherapy was insufficient for patients with CM. Our results might thus suggest that these individuals require more comprehensive interventions. Future research should therefore explore how treatment could be optimized for this particular patient subgroup. Specialized therapies may include adaptations of Cognitive Behavioral Therapy (CBT) aimed at addressing early life adversity and social skills (Miniati et al., 2010), imagery rescripting for depression with intrusive memories (Brewin et al., 2009), and the Cognitive Behavioral Analysis System of Psychotherapy (CBASP) (McCullough, 2003). Additional therapeutic options could include Compassionate Mind Training (Gilbert & Procter, 2006), mindful self-compassion training (Germer & Neff, 2019), and transdiagnostic group compassion-focused therapy (Riebel & Weiner, 2023). Given the serious long-term consequences of CM, increased investment in therapeutic strategies is warranted (Gilbert et al., 2009).

This study highlights the significance of the CTQ for predictions in a real-word setting despite some limitations despite differences in retrospective and prospective measures of CM and compared to the Maltreatment and Abuse Chronology of Exposure (MACE) Scale. While the MACE scale involves a structured interview, the CTQ gathers self-reported information about maltreatment experiences up to age 18. To enhance the precision in distinguishing CM, subsequent investigations could employ distinct patterns (Goerigk et al., 2023). The CTQ provides consistently stable self-reports of CM, regardless of current depressive symptomatology (Goltermann et al., 2023). With its short administration duration (Wingenfeld et al., 2010), the CTQ holds the potential for efficiently identifying individuals at risk for recurrent and persistent forms of depression within clinical settings (Nanni, Uher, & Danese, 2012). It is crucial for clinicians to recognize that routine inquiries into CM bear no inherent harm (Becker-Blease & Freyd, 2006) and can contribute valuable prognostic insights to diagnostic evaluations.

The article emphasizes the need to integrate CM assessment into clinical practice for improved patient care, personalized treatment strategies, and a comprehensive understanding of the intricate relationship between childhood maltreatment and depressive disorders.

### Limitations

Effects observed in nearly all analyses, except for depression severity, exhibited small effect sizes, aligning with other health predictors like smoking and obesity (Rutledge & Loh, 2004). This expected outcome reflects the multifaceted interplay of genetic, environmental, and non-specific factors influencing mental health and clinical parameters (Cuijpers et al., 2012). The reduced sample size for analyzing symptom trajectories during inpatient treatment within a subset of the CRS may constrain the statistical power of the linear mixed models.

The CTQ measures a history of CM, without distinguishing between experiences in different phases of childhood and young adulthood. Future investigations should employ maltreatment assessment tools capable of discerning variations across different age groups, allowing for a more comprehensive understanding of critical time periods.

The presence of substantial heterogeneity both within and between the samples, particularly in terms of comorbidities, warrants careful consideration when interpreting the outcomes while using depression severity as a conventional metric for overarching psychopathology (Piotrowski, 1996). Our analyses might not fully encompass variations or shifts in psychopathology stemming from comorbid disorders.

This study faced time constraints due to divergent follow-up periods, assessing the CRS after 1 year and the CoSS after 2 years, influencing the interpretation of our findings. Additionally, 1- and 2-year intervals encompass a relatively brief period within the broader scope of an individual’s lifespan, implying subsequent investigations with more extensive time intervals.

The considerable dropout in both samples (CRS: 43.84%, CoSS: 22.22%), as a common challenge in longitudinal studies, could lead to bias and diminished statistical power.

## Supporting information

Ratzsch et al. supplementary material Ratzsch et al. supplementary material

## Data Availability

The data that support the findings of this study are not publicly available due to being highly sensitive patient data. The corresponding author can be contacted for further information.

## Supplementary material

The supplementary material for this article can be found at http://doi.org/10.1017/S0033291725000984.
