# Supplementary material for: Childhood maltreatment’s influence on the dynamic course of depression: symptom trajectories during inpatient treatment and after discharge
Source: Psychol Med. 2025 May 2;55:e127. doi: 10.1017/S0033291725000984 (PMC12094660; doi:10.1017/S0033291725000984)
Supplement: Ratzsch et al. supplementary material [file S0033291725000984sup001.docx]

**Supplements**

**Supplementary Methods**

*Clinical Routine Sample (CRS)*

For our study, we selected a sample (clinical routine sample) of *n* = 438 participants diagnosed with Major Depressive Disorder (MDD) during an acute depressive episode, of which 54.57% identified as female (age mean 39.34 ± 16.84). Follow-up assessments were conducted with *n* = 246 participants (56% female, age mean 40.5 ± 16.94) after one year.

The clinical routine sample (CRS) data were sourced from the IZKF SEED 11/18 study, a longitudinal naturalistic examination that endeavors to enhance standard clinical documentation by incorporating digital data collection within the scope of inpatient psychiatric treatment (Richter et al., 2020).

Ethical clearance was obtained from the local Institutional Review Board (IRB) and participants provided informed consent prior to their involvement. Financial compensation was not provided to participants for their engagement.

Patient recruitment was conducted among individuals admitted to the inpatient service of the Department of Psychiatry at the University of Münster during the assessment period spanning from March 2019 to March 2021. Exclusion criteria were only sufficient mental stability and proficiency in reading and writing German. The ‘wide’ inclusion criteria were made to ensure a robust representation of the authentic population seeking psychiatric inpatient treatment.

*Cohort Study Sample (*C*oSS):*

The data utilized in the cohort study sample were derived from two comparable neuroimaging studies. The CoSS sample comprised participants diagnosed with MDD during an acute depressive episode, resulting in an ultimate count of *n* = 567 participants, of which 55.38% were female (age mean 37.59 ± 12.83). Follow-up assessments were conducted with *n* = 441 participants (56% female, age mean 38.72 ± 13.13) after two years.

The first study within the CoSS is the ongoing Marburg-Münster Affective Disorders Cohort Study (MACS consortium) (Kircher et al., 2019). Participants were drawn from individuals seeking treatment at the inpatient facilities of the Universities of Marburg and Münster, Germany. Initial recruitment was from September 2014 to September 2018. In addition to an extensive battery of neuroimaging assessments, a comprehensive neuropsychological evaluation battery was administered. Alongside self-report questionnaires, participants underwent symptom-specific external evaluations and the Structured Clinical Interview for DSM-IV (SCID-I) (Wittchen et al., 1997).

The second study, the Münster Neuroimaging Cohort, was conducted at the Department of Psychiatry, University of Münster, Germany. Initial recruitment was from March 2010 to January 2016. Similar to the MACS Study, all participants underwent structural MRI at the time of primary recruitment and clinical assessments using structured interviews based on DSM-IV criteria were carried out at baseline. Additional information about the study sample can be found in previous publications (Opel et al., 2019; Redlich et al., 2016; Zaremba et al., 2018).

For both neuroimaging studies, participants were invited to participate in a follow-up assessment after two years. Ethics approval for these studies was granted by the local Institutional Review Board (IRB), with all participants providing their informed written consent before partaking. Financial compensation was extended to participants for their involvement. Patients with verbal IQ < 80, history of head trauma or unconsciousness, current intake of benzodiazepines, and neurological illness were excluded from the study.

Measures

The German version of the 28-item Childhood Trauma Questionnaire (CTQ) (Wingenfeld et al., 2010) was administered to assess CM experienced before age 18. The CTQ is a retrospective self-report questionnaire assessing emotional neglect, physical neglect, emotional abuse, physical abuse and sexual abuse, as well as a minimization-denial subscale (Bernstein et al., 1998). The sum of subscale scores can be used to represent an overall maltreatment load, with higher scores indicating higher maltreatment.

For the retrospective clinical analyses, the utilized variables were the number of hospitalizations and age at the first psychiatric or psychotherapeutic treatment, based on a self-report. In assessing the progression of depression severity across various timepoints, the primary metric utilized was the cumulative score derived from the Beck Depression Inventory (BDI). This assessment tool was applied at multiple time points during the inpatient therapeutic process and subsequent follow-up. The BDI, a 21-item self-report questionnaire introduced by Beck et al. (1961), was adopted to precisely evaluate variations in symptomatology (Titov et al., 2011).

Similarly, depression severity at baseline and follow-up was evaluated using the Hamilton Depression Scale (HAMD-17) (Hamilton, 1967), a widely recognized observer-rated assessment tool. Baseline and follow-up assessments were conducted across both sample groups. In the CRS, an additional HAMD-17 assessment was administered at the conclusion of therapy. We conducted analyses on HAMD-17 to ensure consistency in depression measures. We employed the Global Assessment of Functioning scale (GAF) (Hall, 1995) to assess overall functioning along a continuum from psychological distress to well-being. The survey times for GAF were the same as those for the HAMD-17, ensuring consistency in data collection across the study.

Furthermore, exclusively within the CRS, we incorporated three additional BDI measurements at approximately 14-day intervals. These scores were utilized to investigate the trajectory of therapy and determining response rates, defined as a ≥ 50% reduction in symptoms from baseline (Keller, 2003), assessed at the two-week and four-week marks. Additionally, we employed the BDI and the HAMD-17 at the conclusion of the treatment phase to calculate remission rates. Remission was defined as a BDI score of ≤ 12 and a HAMD-17 score of ≤ 6 (Riedel et al., 2010).

To investigate clinical trajectories after discharge, the Life Chart Interview, a rater-based measure was employed. This rater-based measure that includes a visual timeline, serves as a representation of the participant's mental health journey, such as the onset and duration of each depressive episode within the follow-up period. As outlined in our earlier studies (Zaremba et al., 2018; Opel et al., 2019), DSM-IV criteria were applied to identify depressive episodes and to evaluate relapse within the follow-up period and establish full remission, defined as the absence of symptoms for a minimum of two months, which was required before diagnosing a new episode. Additional observations can be made regarding symptom trajectories after discharge, classified into distinct categories: healthy, one depressive episode with full remission, one depressive episode with partial remission, several depressive episodes with full remission, several depressive episodes with partial remission, dysthymia, and persistent depressive episode.

**Supplementary Results**

Individuals with higher CTQ scores were significantly more likely to *relapse* during the follow-up period, as compared to those with lower CTQ scores across both samples (CRS: *B* = 0.005, *SE* = 0.002; CoSS: *B* = 0.007, *SE* = 0.002) (see Figure 3A and Table S6). The odds ratio in both samples indicated that each additional point in the CTQ score corresponds to a 0.5% increase in the risk of relapse in the CRS and a 0.7% increase in the CoSS. Notably, the effect sizes associated with all CTQ subscales exhibited a consistent pattern (see Table S10).

Subsequent analyses pertain to distinct symptom trajectories. The CTQ score was a predictive factor for determining whether patients achieved *full remission* at the follow-up period in both samples (CRS: *B* = -0.005, *SE* = 0.002; CoSS: *B* = -0.008, *SE* = 0.002) (see Fig. 3B and Table S6). The odds ratio in both samples indicated that each additional point in the CTQ score corresponds to a 0.5% decrease in achieving full remission in the CRS in the one-year follow-up period and a 0.8% decrease in the CoSS in the two-year follow-up period. In both samples, patient subgroups characterized by distinct symptom trajectories, as determined by the Life Chart Interview during the follow-up period, showed significant differences in their CTQ values (CRS: *F*(1, 254) = 4.739, *p* = .030; CoSS: *F*(1, 166) = 15.2, *p* < .001). In short, we observed a consistent pattern of higher CTQ values in patients with more severe, more frequent, or prolonged periods of depressive symptoms. For comprehensive results pertaining to all distinct symptom trajectories, please refer to Table S11.

Additional sensitivity analyses revealed that CTQ score was a significant predictor for BDI during the follow-up intervals (CRS: one-year and CoSS two-year follow-up interval) in both samples (CRS: *B* = 0.723, *SE* = 0.092; CoSS: *B* = 0.583, *SE* = 0.138) (see Table S7).

Moreover, CTQ score emerged as a significant predictor of the duration of depressive episodes during the follow-up period (CRS: *B* = 0.039, *SE* = 0.017; CoSS: *B* = 0.005, *SE* = 0.004) (see Table S7).

**Supplementary Tables**

**Table S1.** Overview of regression analyses predicting number of hospitalizations, age of first psychiatric or psychotherapeutic treatment and BDI prior to inpatient treatment in CRS and CoSS by the CTQ score, all analyses controlled for gender and age.

| Sample | *Outcome* | *Predictor* | *F* | *R²* | *Partial R²* | *B* | *SE B* | *p-value* | *P_FDR_* |
| --- | --- | --- | --- | --- | --- | --- | --- | --- | --- |
| CRS | Number of hospital stays | *Model* | 17.88 | 0.116 |  |  |  | < 0.0001 | < .001 |
|  |  | CTQ |  |  | 0.032 | 0.040 | 0.010 | < 0.0001 | < .001 |
|  | Age of first psychiatric treatment | *Model* | 231.4 | 0.629 |  |  |  | < 0.0001 | < .001 |
|  |  | CTQ |  |  | 0.009 | -0.091 | 0.029 | 0.002 | 0.003 |
|  | BDI | *Model* | 25.49 | 0.160 |  |  |  | < 0.0001 | < .001 |
|  |  | CTQ |  |  | 0.125 | 0.237 | 0.031 | < 0.0001 | < .001 |
|  | Length of inpatient treatment | *Model*  CTQ | 1.173 | 0.017 | 0.002 | -0.014 | 0.096 | 0.321  0.881 | 0.354  0.881 |
| CoSS | Number of hospital stays | *Model* | 28.84 | 0.140 |  |  |  | < 0.0001 | < .001 |
|  |  | CTQ |  |  | 0.056 | 0.036 | 0.006 | <0.0001 | < .001 |
|  | Age of first psychiatric treatment | *Model* | 312.6 | 0.642 |  |  |  | < 0.0001 | < .001 |
|  |  | CTQ |  |  | 0.034 | -0.139 | 0.019 | < 0.0001 | < .001 |
|  | BDI | *Model* | 22.28 | 0.112 |  |  |  | < 0.0001 | < .001 |
|  |  | CTQ |  |  | 0.089 | 0.183 | 0.025 | < 0.0001 | < .001 |

*Note.* CTQ = Childhood Maltreatment Questionnaire, BDI = Beck´s Depression Inventory, CRS = Clinical Routine Sample, CoSS = Cohort Study Sample.

**Table S2.** Overview of regression analyses predicting HAMD and GAF at Baseline and at Follow Up with CTQ in CRS and CoSS, all analyses controlled for age and gender.

| Sample | *Outcome* | *Predictor* | *F* | *R²* | *B* | *SE B* | *p-value* | *P_FDR_* |
| --- | --- | --- | --- | --- | --- | --- | --- | --- |
| CRS | HAM-D  Baseline | *Model* | 11.87 | 0.086 |  |  | < 0.0001 | < .001 |
|  |  | CTQ |  |  | 0.090 | 0.020 | < 0.0001 | < .001 |
|  | HAM-D  Post | *Model* | 5.066 | 0.061 |  |  | 0.002 | 0.003 |
|  |  | CTQ |  |  | 0.123 | 0.032 | 0.0001 | < .001 |
|  | GAF Baseline | *Model*  CTQ | 5.134 | 0.039 | -0.10 | 0.030 | < 0.0001  < 0.001 | < .001  0.002 |
|  | GAF  Post | *Model*  CTQ | 4.204 | 0.051 | -0.147 | 0.045 | 0.006  0.001 | 0.008  0.002 |
| CoSS | HAM-D Baseline | *Model* | 7.09 | 0.034 |  |  | < 0.0001 | < .001 |
|  |  | CTQ |  |  | 0.068 | 0.021 | 0.001 | 0.002 |
|  | HAM-D Post | *Model* | 14.84 | 0.148 |  |  | < 0.0001 | < .001 |
|  |  | CTQ |  |  | 0.158 | 0.025 | < 0.0001 | < .001 |
|  | GAF Baseline | *Model*  CTQ | 9.75 | 0.052 | -0.095 | 0.024 | < 0.0001  < 0.0001 | < .001  < .001 |
|  | GAF Post | *Model*  CTQ | 3.295 | 0.033 | -0.427 | 0.173 | 0.021  0.014 | 0.027  0.019 |

*Note.* HAM-D = Hamilton Rating Scale for Depression, GAF = General Assessment of Functioning scale, CTQ = Childhood Maltreatment Questionnaire, CRS = Clinical Routine Sample, CoSS = Cohort Study Sample.

**Table S3.** Overview of regression analyses predicting number of hospitalizations, age of first psychiatric or psychotherapeutic treatment and BDI prior to inpatient treatment as well as BDI after 2 years in CoSS by the CTQ score, all analyses controlled for gender, age and site.

| Sample | *Outcome* | *Predictor* | *F* | *R²* | *Partial R²* | *B* | *SE B* | *p-value* | *P_FDR_* |
| --- | --- | --- | --- | --- | --- | --- | --- | --- | --- |
| CoSS | Number of hospital stays | *Model* | 21.6 | 0.140 |  |  |  | < 0.0001 | < .001 |
|  |  | CTQ |  |  | 0.056 | 0.036 | 0.006 | <0.0001 | < .001 |
|  | Age of first psychiatric treatment | *Model* | 234.9 | 0.641 |  |  |  | < 0.0001 | < .001 |
|  |  | CTQ |  |  | 0.559 | -0.139 | 0.019 | < 0.0001 | < .001 |
|  | BDI Baseline | *Model* | 29.17 | 0.181 |  |  |  | < 0.0001 | < .001 |
|  |  | CTQ |  |  | 0.091 | 0.182 | 0.024 | < 0.0001 | < .001 |
|  | BDI Post | *Model* | 13.08 | 0.169 |  |  |  | < 0.0001 | < .001 |
|  |  | CTQ |  |  | 0.116 | 0.271 | 0.044 | < 0.0001 | < .001 |

*Note.* CTQ = Childhood Maltreatment Questionnaire, BDI = Beck´s Depression Inventory, CoSS = Cohort Study Sample.

**Table S4.** Overview of regression analyses predicting baseline BDI from physical neglect, physical abuse, emotional neglect, emotional abuse and sexual abuse in CRS and CoSS, all analyses controlled for age and gender.

| Sample | *Predictor* | *F* | *R²* | *B* | *SE B* | *p-value* | *P_FDR_* |
| --- | --- | --- | --- | --- | --- | --- | --- |
| CRS | *Model* | 26.33 | 0.162 |  |  | < 0.0001 | < .001 |
|  | Physical Neglect |  |  | 0.723 | 0.092 | < 0.0001 | < .001 |
|  | *Model* | 14.33 | 0.095 |  |  | < 0.0001 | < .001 |
|  | Physical Abuse |  |  | 0.774 | 0.149 | < 0.0001 | < .001 |
|  | *Model* | 20.13 | 0.130 |  |  | < 0.0001 | < .001 |
|  | Emotional Neglect |  |  | 0.594 | 0.090 | < 0.0001 | < .001 |
|  | *Model* | 26.33 | 0.162 |  |  | < 0.0001 | < .001 |
|  | Emotional Abuse |  |  | 0.723 | 0.092 | < 0.0001 | < .001 |
|  | *Model* | 7.19 | 0.050 |  |  | < 0.0001 | < .001 |
|  | Sexual Abuse |  |  | 0.395 | 0.151 | 0.009 | 0.012 |
| CoSS | *Model* | 10.23 | 0.054 |  |  | < 0.0001 | < .001 |
|  | Physical Neglect |  |  | 0.583 | *0.138* | < 0.0001 | < .001 |
|  | *Model* | 11.57 | 0.061 |  |  | < 0.0001 | < .001 |
|  | Physical Abuse |  |  | 0.535 | 0.116 | < 0.0001 | < .001 |
|  | *Model* | 18.62 | 0.095 |  |  | < 0.0001 | < .001 |
|  | Emotional Neglect |  |  | 0.491 | 0.075 | < 0.0001 | < .001 |
|  | *Model* | 26.16 | 0.129 |  |  | < 0.0001 | < .001 |
|  | Emotional Abuse |  |  | 0.618 | 0.077 | < 0.0001 | < .001 |
|  | *Model* | 7.71 | 0.042 |  |  | < 0.0001 | < .001 |
|  | Sexual Abuse |  |  | 0.389 | 0.122 | 0.002 | 0.003 |

*Note.* BDI = Beck’s Depression Inventory, CRS = Clinical Routine Sample, CoSS = Cohort Study Sample.

**Table S5.** Overview of regression analyses predicting HAMD and GAF at Baseline and at Follow Up with CTQ in CoSS, all analyses controlled for age, gender and site.

| Sample | *Outcome* | *Predictor* | *F* | *R²* | *B* | *SE B* | *p-value* | *P_FDR_* |
| --- | --- | --- | --- | --- | --- | --- | --- | --- |
| CoSS | HAM-D Baseline | *Model* | 105 | 0.447 |  |  | < 0.0001 | < .001 |
|  |  | CTQ |  |  | 0.077 | 0.016 | < 0.0001 | < .001 |
|  | HAM-D Post | *Model* | 14.64 | 0.186 |  |  | < 0.0001 | < .001 |
|  |  | CTQ |  |  | 0.179 | 0.026 | < 0.0001 | < .001 |
|  | GAF Baseline | *Model*  CTQ | 14.12 | 0.096 | -0.094 | 0.023 | < 0.0001  < 0.0001 | < .001  < .001 |
|  | GAF Post | *Model*  CTQ | 14.32 | 0.167 | -0.458 | 0.161 | < 0.0001  0.005 | < .001  0.007 |

*Note.* HAM-D = Hamilton Rating Scale for Depression, GAF = General Assessment of Functioning scale, CTQ = Childhood Maltreatment Questionnaire, CoSS = Cohort Study Sample.

**Table S6.** Overview of the Linear Mixed Model results for changes in BDI during inpatient treatment in the CRS with CM, time in Model A and additionally with the interaction of CM and time in Model B as predictors, analyses controlled for gender and age.

| Model | *Predictor* | *Estimate* | *SE* | 95% *CI* | *t-value* | *p-value* | *P_FDR_* |
| --- | --- | --- | --- | --- | --- | --- | --- |
| Model A | Intercept  CTQ  Time | 18.344  0.177  -0.159 | 3.395  0.065  0.022 | [11.556, 25.143]  [0.046, 0.307]  [-0.203, -0.115] | 5.403  2.710  -7.135 | <0.001  0.008  <0.001 | 0.002  0.011  0.002 |
| Model B | Intercept  CTQ  Time  CTQ * Time | 16.933  0.207  -0.061  -0.002 | 3.535  0.068  0.071  0.001 | [9.942, 23.934]  [0.071, 0.342]  [-0.202, 0.081]  [-0.005, 0.000] | 4.790  3.018  -0.845  -1.455 | <0.001  0.003  0.400  0.152 | 0.002  0.004  0.425  0.171 |

*Note.* CTQ = Childhood Maltreatment Questionnaire.

**Table S7.** Overview of linear mixed models predicting *course of therapy* (based on baseline and three additional BDI measurements) from physical neglect, physical abuse, emotional neglect, emotional abuse and sexual abuse in CRS, all analyses controlled for age and gender.

| *Predictor* | *Estimate* | *SE* | *t-value* | *p-value* | *P_FDR_* |
| --- | --- | --- | --- | --- | --- |
| Intercept  Physical Neglect  Time  Physical Neglect * Time | 20.905  0.484  -0.096  -0.005 | 2.846  0.205  0.054  0.004 | 7.346  2.362  -1.791  -1.263 | < 0.001  0.020  0.077  0.210 | 0.002  0.026  0.089  0.230 |
| Intercept  Physical Abuse  Time  Physical Abuse * Time | 21.187  0.838  -0.047  -0.016 | 2.713  0.353  0.955  0.007 | 7.811  2.370  -0.863  -2.226 | < 0.001  0.020  0.390  0.028 | 0.002  0.026  0.417  0.035 |
| Intercept  Emotional Neglect  Time  Emotional Neglect * Time | 19.452  0.529  -0.106  -0.004 | 2.844  0.180  0.060  0.004 | 6.838  2.940  -1.780  -0.955 | < 0.001  0.004  0.078  0.342 | 0.002  0.006  0.090  0.369 |
| Intercept  Emotional Abuse  Time  Emotional Abuse * Time | 20.905  0.484  -0.097  -0.005 | 2.846  0.205  0.054  0.004 | 7.346  2.362  -1.791  -1.263 | < 0.001  0.020  0.077  0.210 | 0.002  0.026  0.089  0.230 |
| Intercept  Sexual Abuse  Time  Sexual Abuse * Time | 24.890  0.268  -0.085  -0.011 | 2.920  0.335  0.046  0.006 | 8.523  0.798  -1.846  -1.844 | < 0.001  0.427  0.068  0.069 | 0.002  0.446  0.081  0.081 |

*Note.* CRS = Clinical Routine Sample.

**Table S8.** Logistic regressions on response and remission rates with childhood maltreatment as a predictor in the CRS, all analyses controlled for gender and age.

| Sample | *Outcome* | *Predictor* | *R²* | *B* | *SE B* | *p-value* | *P_FDR_* | *Odds ratio* |
| --- | --- | --- | --- | --- | --- | --- | --- | --- |
| CRS | Response after 2 weeks | *Model* | 0.222 |  |  |  |  |  |
|  |  | CTQ |  | -0.004 | 0.001 | < 0.001 | 0.002 | 0.996 |
|  |  | *Model*  CTQ  BDI pre | 0.223 | -0.003  -0.004 | 0.001  0.002 | 0.014  0.054 | 0.019  0.065 | 0.997  0.996 |
|  | Response after 4 weeks | *Model* | 0.280 |  |  |  |  |  |
|  |  | CTQ |  | -0.001 | 0.002 | 0.659 | 0.664 | 0.999 |
|  |  | *Model*  CTQ  BDI pre | 0.304 | 0.001  -0.010 | 0.002  0.003 | 0.491  0.002 | 0.505  0.003 | 1.001  0.991 |
|  | Remission  BDI | *Model*  CTQ | 0.579 | -0.006 | 0.002 | 0.004 | 0.006 | 0.994 |
|  |  | *Model*  CTQ  BDI pre | 0.628 | -0.0004  -0.020 | 0.002  0.003 | 0.825  < 0.0001 | 0.825  < .001 | 1.000  0.980 |

*Note*. CTQ = Childhood Maltreatment Questionnaire, BDI pre = Beck’s Depression Inventory at Baseline, CRS = Clinical Routine Sample.

**Table S9.** Overview of logistic regressions predicting remission concerning HAM-D at discharge of inpatient treatment with CTQ as a predictor in CRS, further controlled for HAM-D at Baseline.

| Sample | *Outcome* | *Predictor* | *R²* | *B* | *SE B* | *p-value* | *P_FDR_* |
| --- | --- | --- | --- | --- | --- | --- | --- |
| CRS | Remission HAM-D | *Model*  CTQ | 0.628 | -0.095 | 0.052 | < 0.0001  < 0.0001 | < .001  < .001 |
|  |  | *Model*  CTQ  HAM-D Baseline | 0.719 | -0.001  -0.030 | 0.002  0.005 | 0.567  < 0.0001 | 0.575  < .001 |

*Note.* HAM-D = Hamilton Rating Scale for Depression, CTQ = Childhood Maltreatment Questionnaire, CRS = Clinical Routine Sample.

**Table S10.** Overview of logistic regressions on relapse rates and achieving full remission with childhood maltreatment as a predictor in CRS after one year and in CoSS after two years, all analyses controlled for gender and age.

| Sample | *Outcome* | *Predictor* | *B* | *SE B* | *p-value* | *P_FDR_* | *Odds ratio* |
| --- | --- | --- | --- | --- | --- | --- | --- |
| CRS | Relapse | *Model* |  |  |  |  |  |
|  |  | CTQ | 0.005 | 0.002 | 0.002 | 0.003 | 1.005 |
|  | Full remission | *Model*  CTQ | -0.005 | 0.002 | 0.017 | 0.023 | 0.995 |
| CoSS | Relapse | *Model* |  |  |  |  |  |
|  |  | CTQ | 0.007 | 0.002 | 0.003 | 0.004 | 1.007 |
|  | Full remission | *Model*  CTQ | -0.008 | 0.002 | < 0.001 | 0.002 | 0.992 |

*Note*. CTQ = Childhood Maltreatment Questionnaire, CRS = Clinical Routine Sample, CoSS = Cohort Study Sample.

**Table S11.** Overview of logistic regression analyses predicting *Relapse* from physical neglect, physical abuse, emotional neglect, emotional abuse and sexual abuse in CRS and CoSS, all analyses controlled for age and gender.

| Sample | *Predictor* | *B* | *SE B* | *p-value* | *P_FDR_* | *Odds ratio* |
| --- | --- | --- | --- | --- | --- | --- |
| CRS | *Model* |  |  | < 0.0001 | < .001 |  |
|  | Physical Neglect | 0.011 | 0.005 | 0.034 | 0.042 | 1.011 |
|  | *Model* |  |  | < 0.0001 | < .001 |  |
|  | Physical Abuse | 0.017 | 0.009 | 0.050 | 0.061 | 1.017 |
|  | *Model* |  |  | < 0.0001 | < .001 |  |
|  | Emotional Neglect | 0.015 | 0.005 | 0.002 | 0.003 | 1.015 |
|  | *Model* |  |  | < 0.0001 | < .001 |  |
|  | Emotional Abuse | 0.011 | 0.005 | 0.034 | 0.042 | 1.011 |
|  | *Model* |  |  | < 0.0001 | < .001 |  |
|  | Sexual Abuse | 0.023 | 0.008 | 0.003 | 0.004 | 1.024 |
| CoSS | *Model* |  |  | 0.002 | 0.003 |  |
|  | Physical Neglect | 0.009 | 0.011 | 0.419 | 0.441 | 1.009 |
|  | *Model* |  |  | < 0.001 | 0.002 |  |
|  | Physical Abuse | 0.007 | 0.010 | 0.485 | 0.503 | 1.007 |
|  | *Model* |  |  | 0.004 | 0.006 |  |
|  | Emotional Neglect | 0.008 | 0.006 | 0.161 | 0.179 | 1.008 |
|  | *Model* |  |  | 0.002 | 0.003 |  |
|  | Emotional Abuse | 0.010 | 0.006 | 0.113 | 0.129 | 1.010 |
|  | *Model* |  |  | < 0.001 | 0.002 |  |
|  | Sexual Abuse | 0.009 | 0.009 | 0.334 | 0.363 | 1.009 |

*Note.* CRS = Clinical Routine Sample, CoSS = Cohort Study Sample.

**Table S12.** Overview of logistic regressions on relapse rates and achieving full remission with childhood maltreatment as a predictor in CoSS after two years, all analyses controlled for gender, age and site.

| Sample | *Outcome* | *Predictor* | *B* | *SE B* | *p-value* | *P_FDR_* | *Odds ratio* |
| --- | --- | --- | --- | --- | --- | --- | --- |
| CoSS | Relapse | *Model* |  |  |  |  |  |
|  |  | CTQ | 0.007 | 0.002 | 0.003 | 0.004 | 1.007 |
|  | Full remission | *Model*  CTQ | -0.008 | 0.002 | < 0.001 | 0.002 | 0.992 |

*Note*. CTQ = Childhood Maltreatment Questionnaire, CoSS = Cohort Study Sample.

**Table S13.** Descriptive Statistics for CTQ in distinct symptom trajectories after discharge.

| Symptom Trajectory | Sample | *M* | *SD* |
| --- | --- | --- | --- |
| healthy mood  one depressive episode with complete remission  one depressive episode with partial remission  several depressive episodes with complete remission  dysthymic course  several depressive episodes with partial remission  persistent | CRS  CoSS  CRS  CoSS  CRS  CoSS  CRS  CoSS  CRS  CoSS  CRS  CoSS  CRS  CoSS | 41.57  42.02  47.97  46.96  49.37  50.00  48.23  44.04  47.77  49.31  48.33  39.50  50.48  60.25 | 12.98  15.46  20.73  16.62  15.04  16.38  17.58  11.58  16.20  19.01  15.55  7.85  19.14  12.36 |

*Note.* CRS = Clinical Routine Sample, CoSS = Cohort Study Sample.

**Table S14.** Overview of linear regressions on BDI Post and duration of depressive episodes with CTQ as a predictor in CRS after one year and in CoSS after two years, all analyses controlled for gender and age.

| Sample | *Outcome* | *Predictor* | *F* | *R²* | *B* | *SE B* | *p-value* | *P_FDR_* |
| --- | --- | --- | --- | --- | --- | --- | --- | --- |
| CRS | BDI | *Model* | 5.429 | 0.093 |  |  | 0.001 | 0.002 |
|  |  | CTQ |  |  | 0.243 | 0.061 | < 0.0001 | < .001 |
|  | Duration of depressive episodes | *Model* | 3.225 | 0.095 |  |  | 0.024 | 0.030 |
|  |  | CTQ |  |  | 0.039 | 0.017 | 0.021 | 0.027 |
| CoSS | BDI | *Model* | 14.26 | 0.142 |  |  | < 0.0001 | < .001 |
|  |  | CTQ |  |  | 0.266 | *0.045* | < 0.0001 | < .001 |
|  | Duration of depressive episodes | *Model* | 0.801 | 0.008 |  |  | 0.494 | 0.505 |
|  |  | CTQ |  |  | 0.005 | 0.004 | 0.146 | 0.165 |

*Note*. CTQ = Childhood Maltreatment Questionnaire, CRS = Clinical Routine Sample, CoSS = Cohort Study Sample.

**References Supplementary Material**

Titov, N., Dear, B. F., McMillan, D., Anderson, T., Zou, J., & Sunderland, M. (2011). Psychometric comparison of the PHQ-9 and BDI-II for measuring response during treatment of depression. *Cognitive behaviour therapy*, *40*(2), 126–136.

Wittchen, H.-U., Zaudig, M., & Fydrich, T. (1997). *Skid. Strukturiertes klinisches Interview für DSM-IV. Achse I und II. Handanweisung*.
